# Understanding the impact of triazoles on female fertility and embryo development: Mechanisms and implications

**DOI:** 10.1016/j.toxrep.2025.101948

**Published:** 2025-02-04

**Authors:** Sonal Sharma, Geeta Pandey

**Affiliations:** Department of Zoology, IIS (deemed to be University), Jaipur, Rajasthan 302020, India

**Keywords:** Toxicity, Triazole, Fungicides, Fertility, Teratogenicity

## Abstract

Triazoles are among the most widely used fungicides that were launched in 1980s and are one of the most important pesticide groups used in agriculture as plant growth regulators and stress protectors. Triazoles are also frequently used in the pharmaceutical industry to treat fungal and bacterial infections as well as to treat and prevent some forms of pneumonia. Humans are normally exposed to triazoles through food, water, and medications, which raises concerns about their potential adverse effects on health. Therefore, this review was planned to examine the impact of triazole fungicides on female fertility, as well as their teratogenic and embryotoxic effects. Various search engines such as PubMed, Google Scholar, Elsevier, IEEE were used to search the relevant articles published between 2006 and 2024 using the following keywords: "azoles," "female infertility," "reproductive toxicity," "teratogenicity," "triazoles," and "embryo toxicity." The findings suggest that triazoles might negatively affect female fertility and embryonic development through multiple mechanisms including inhibition or interference with key enzymes such as CYP17A1 and CYP19A1 (aromatase) involved in steroid hormone synthesis, endocrine disruption, oxidative stress, disruption of signaling pathways, and apoptosis. This review consolidates current knowledge on the teratogenic and embryotoxic properties of triazole fungicides, providing a comprehensive understanding of their health implications and addressing critical research gaps.

## Highlights

• Triazoles exposure in humans can occur through agriculture, healthcare, and antifungal medications.
• Triazoles impair ovarian function, follicular development, ovulation, and oocyte maturation, reducing fertility.
• Prolonged exposure to triazoles disrupts early embryonic development and may cause teratogenic effects.
• Triazoles inhibit cytochrome P450 enzymes, disrupting estrogen production and hormonal balance in females.

## Graphical Abstract

**Figure:**
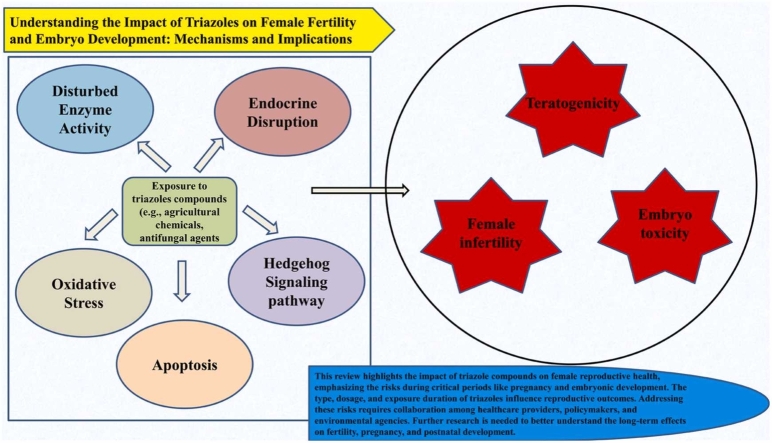

## 1 Introduction

Azoles are a diverse and important class of organic compounds distinguished by the presence of a five-membered ring containing at least one nitrogen atom. This heterocyclic structure gives them a wide range of chemical and biological capabilities, making them useful in a variety of sectors like as medicines, agriculture, and materials research. Azoles are categorized in two categories: triazoles and imidazoles. Both of them are being abundantly used in medicinal chemistry to treat fungal and bacterial diseases, in organic chemistry to synthesize complicated compounds and in horticulture to limit fungus growth on trees, grasses, vegetables, seeds, and fruits. Some azoles are also used in wood preservation to prevent deterioration and fungal growth, hence increasing the lifespan of wood goods [1].

Triazoles represent the second-largest class of fungicides globally. These fungicides have received a lot of interest because of their diverse applications and importance in a variety of industries. They are widely utilized in agriculture to treat a number of fungal diseases, including rust, powdery mildew, and leaf-spotting fungi, affecting crops like fruits, vegetables, ornamentals, and grains [2], [3]. Tetraconazole Tebuconazole and propiconazole are some of the commonly used triazoles in agriculture to protect crops against fungal infections. Triazoles exert antifungal effects by competitively inhibiting the enzyme (cytochrome P51) CYP51 (lanosterol-14α-demethylase), which is essential for sterol production in fungi. This specific inhibition depletes ergosterol while causing an accumulation of lanosterol and other 14-methyl sterols, resulting to changes in the fungal cell membrane and eventually suppressing fungal development [4], [5]. Triaozles also act as vital building blocks in the manufacturing of many medications such as fluconazole and itraconazole, which are used to treat a variety of fungal infections, including yeast infections and systemic infections [6], Ketoconazole and other azole derivatives are used as hormone synthesis inhibitors, particularly in the treatment of conditions such as Cushing's syndrome by inhibiting the synthesis of cortisol and other steroid hormones [7]. Azoles, particularly triazoles and imidazoles also showed great potential as antifouling agents with respect to protection against fouling of barnacles [8].

**Fig. 1:**
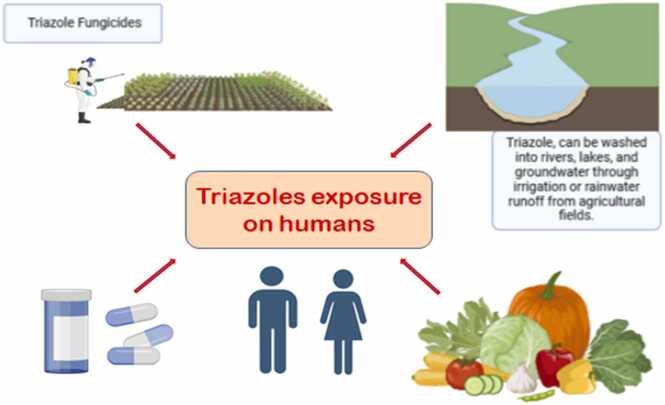
Humans exposure to triazoles Created with BioRender https://www.biorender.com.

Due to its high usage, humans face significant occupational and environmental exposure to triazole fungicides. Triazole fungicides can enter aquatic environments through runoff, agricultural return flows, groundwater infiltration, and plant up take. Consequently, they are frequently detected in aquatic habitats around the world. Humans can get exposed to triazoles through contaminated water sources, food consumption, and environmental exposure. Triazole fungicide residues are commonly found in soil, water, food, and animal feed due to their high chemical stability, low biodegradability, and ability to easily spread through the environment. These residues have been detected in various environmental matrices [9], [10], [11], as well as in food and feed from both plant and animal sources [11], [12]. Additionally, traces of these fungicides have been identified in human urine [13]. This highlights the need to address the potential ecological and health risks posed by triazole fungicides. Triazole compounds, including Propiconazole (PP), Metconazole (MC), Epoxiconazole (Epox), prothioconazole (PT), difenoconazole (Dif), tebuconazole (TB), tetraconazole (TT), and cyproconazole (Cypro), have been implicated in a number of serious health risks for organisms, including endocrine disruption [14], [15], developmental and reproductive toxicity [16], and liver toxicity [17], [18]. Understanding the potential effects of triazole exposure on reproductive health is crucial for identifying the risks to communities living near where these compounds are used. The present review was planned to address the knowledge gaps that persist despite of considerable evidences available regarding adverse impact of triazole fungicides. It will offer a roadmap for future studies aimed at alleviating their risks and improving public health outcomes.

## 2 Effects of triazoles on female fertility and embryonic development

According to WHO (World Health Organization), around 48 million couples and 186 million individuals are facing the problem of infertility worldwide [19]. It is defined as inability to achieve pregnancy after a year of frequent unprotected intercourse. It is a complex disorder with many contributing causes, thus it is critical to consider both male and female components. Ovulatory dysfunction, male factor infertility, and tubal disease are the three most prevalent causes of infertility. Male reproductive health has traditionally received the most attention while discussing endocrine disruptors. In contrast, the early stages of female reproductive development are less reliant on steroid hormone signalling, which accounts for the lack of research on this topic. However, what else could be responsible for the observed connections between foetal exposure to endocrine disruptors and female reproductive issues if it is not due to altered steroid signalling? A clear explanation would be a change in ovarian development, which would eventually result in disease due to a decrease in ovary/endocrine function [20].

Female reproductive health is dependent on normal ovary development, which begins during fetal life. From fetal development through reproductive maturity, the coordinated interaction of oocytes, granulosa cells, theca cells, and other specialized cell types leads to the formation of fully functional ovaries [21], [22]. Environmental toxins can affect individuals throughout the lifespan, including prenatally, and can have various effects from oogenesis to ovulatory dysfunction to abnormal development of embryo. In recent years, there has been a greater focus on how early exposure to environmental contaminants affects female reproductive health later in life. Female reproductive toxicity associated with Triazole exposure has been studied in various animal models, including zebrafish [23], [24], [25], earthworm [26], bird [27], [28], Xenopus laevis [29], [30], and rats [31]. Here, we have summarized the available studies on Triazole exposure on female reproductive organs, fertility and embryo development.

Triazoles might interfere with the natural growth and release of eggs, affecting people's capacity to conceive. Rockett *et al*. [8] reported that administration of Propiconazole from gestation day 6 to postnatal day 98 induced disturbance in estrogen cyclicity and decline in the weight of liver in rats. Gal and Orly [32] investigated the effects of ketoconazole, an antifungal agent, on ovarian steroidogenesis in rat cells and revealed potent inhibitory effects of ketoconazole on ovarian steroidogenesis by selectively inhibiting key enzymes involved in the production of steroid hormones, particularly estrogen and progesterone.

Triazoles have also been found to have teratogenic properties and can induce birth defects or developmental abnormalities in fetuses if they are exposed during critical stages of embryonic development therefore exposure of triazoles during pregnancy is a topic of concern. Pregnant women may be exposed to triazoles, either intentionally or unintentionally, especially if they are undergoing medical treatment containing these compounds. Some triazoles, particularly those used as antifungal medications, have been studied for teratogenicity and the developmental defects [33]. Fluconazole, itraconazole, voriconazole, and posaconazole are among the most commonly used triazole antifungals, which work by inhibiting the synthesis of ergosterol, a key component of fungal cell membranes [34].

In a study, El-Shershaby *et al*. [35] noticed failed pregnancy, fetal resorption and embryo disorganization, alterations in the histology of uterus tissue and expression of TGFβ2, TNFR2, Caspase 10, and HSP70 in pregnant female mice administered penconazole (PEN) daily 30, 20, 10, or 5 mg/kg BW or the day after the other day (5 mg/kg BW). When pregnant females were treated with PEN during pre- implantation period (2.5-mg/kg BW), and during post-implantation period (2.5-mg/kg BW), expression of TGFβ2, TNFR2, Caspase 10, and HSP70 was noted to be upregulated along with altered histological and immunohistochemical analysis of the uterine environment. In another study, El-Shershaby *et al*. [36] noted that higher doses (30, 20, 10, and 5 mg/kg body weight) of penconazole resulted in fetal resorption. Penconazole exposure at a lower dose (2.5 mg/kg) induced developmental delay in the cerebral hemisphere, hippocampus, and cerebellum (lobulation), It caused retinopathy during eye development and reduced the expression of α-synuclein in the hippocampus, cerebellum, and ganglion cell layer of the developing brain and eye.

Exposure of itraconazole at high dose level to various animal models revealed an increased risk of skeletal deformities therefore Itraconazole is often avoided during pregnancy, particularly in the first trimester. El-Sherhaby *et al*. [37] recorded increased teratogenicity during organogenesis, with the most common abnormalities including abdominal hernia, protruding tongue, exencephaly, incompletely ossified, unossified, or missing skull bones (mostly frontal, parietal, and interparietal), abnormal vertebrae, and fused or supernumerary ribs in pregnant female rats orally administered with itraconazole. Itraconazole also increased DNA damage in fetal osteocytes via significant increase in the measured comet parameters in all the treated groups, indicating that itraconazole severely affects fetal genetic material. Itraconazole exposure in pregnant ICR (CD-1) mice revealed various morphological abnormalities such as cleft palate, limb deformities, and axial skeletal malformations in fetus [6].

**Fluconazole** is an antifungal medication commonly used to treat severe fungal infections. Exposure to the fluconazole during the first trimester of pregnancy increases the risk of birth malformations, such as skeletal and cardiac defects. Therefore FDA has recommended that fluconazole should not be used during the first trimester of pregnancy. Tiboni *et al.* [38] treated pregnant mice with a single dosage of fluconazole and embryos were harvested 12, 24, and 48 hours following treatment. Fluconazole exposure induced increased expression of CYP26a1 and CYP26b1, but no significant change was observed in the CYP26c1 isoform which indicated that fluconazole can alter expression of CYP26 gene expression in mouse embryos. Abnormal CYP26 expression can lead to disruptions in retinoic acid signaling, potentially resulting in developmental disorders and other health issues [39]. CYP26 plays a key role in regulating retinoic acid levels in the body and ensure proper developmental processes and cellular functions as Retinoic acid, a derivative of vitamin A, is crucial for numerous physiological processes including cell growth, differentiation, and embryonic development. Similarly Zheng *et al*. [40] noticed an increase in the mRNA expression level of genes implicated in retinoic acid synthesis while significant decrease in genes involved in RA catabolism. Amaral *et al*. [7] recorded malformations in embryos in rats treated with fluconazole and ketoconazole.

Tebuconazole might affect placental development and fetal health via disrupting critical functions of trophoblast cells. When trophoblast cells (BeWo cell line) were exposed to various concentrations of tebuconazole, significant reduction was noted in cell viability and proliferation along with suppression in the secretion of key hormones such as human chorionic gonadotropin (hCG) and pregnancy-associated plasma protein-A (PAPP-A) [41].

Some studies suggest that triazole mixtures may have an impact on the female reproductive system. However, the extent of these effects can vary depending on the specific triazole compound, dosage, exposure duration, and the physiological condition of the organism being studied. Co-exposure to azoles (retinoic acid, flusilazole, triadimefon, and cyproconazole) significantly increased teratogenic effects such as craniofacial malformations as compared to single exposures in rat embryos via disrupting the retinoic acid pathway that can simulate the formation of the physiological retinoic acid gradient in the rat embryo hindbrain [42]. Similar results were obtained by [43] when rat embryos were treated with flusilazole, cyproconazole, triadimefon, all-trans-retinoic acid, or vehicle control dimethyl sulfoxide (DMSO). Menegola *et al.* [44] observed a clear azole-specific dysmorphogenic effect inducing craniofacial and branchial arches deformities following *in vitro* exposure of Triadimefon, Imazalil, Triadimenol, Cyproconazole, Tebuconazole, and Flusilazole to the rat embryo.

De Jong *et al*. [45] assessed the developmental toxicity of six 1,2,4-triazole compounds—flusilazole, hexaconazole, cyproconazole, triadimefon, myclobutanil, and triticonazole—using the mouse Embryonic Stem Cell Test (EST), the Zebrafish Embryotoxicity Test (ZET), and the rat postimplantation Whole Embryo Culture (WEC). The rat postimplantation Whole Embryo Culture revealed a consistent pattern of teratogenic effects, including the reduction and fusion of the first and second branchial arches.

Exposure of prothioconazole, metconazole, difenoconazole, tetraconazole, and cyproconazole (0.001 µM to 1000 µM) for 48 h induced reduction in the secretion of progesterone, estradiol, or both along with decrease in the expression of CYP51, STAR, CYP11A1, CYP19A1, or HSD3B proteins, or a combination thereof, in hGCs (Human granulosa cells) and KGN cells and an increase in aryl hydrocarbon receptor (AHR) nuclear receptor in KGN cells [16].

Shen *et al*. [46] exposed zebrafish embryos to 0.850 mg/L prothioconazole and noted adverse impact on embryo survival and hatching. Exposure to prothioconazole resulted in embryonic malformations, particularly affecting the yolk sac and causing pericardial edema. In addition, prothioconazole- induced apoptosis by up-regulating the expression of oxidative stress defense-related genes and p53 and via increasing ratio of bax to bcl2 in time and concentration dependent manner.

Rieke et al. [47] investigated the combined effects of triazole fungicides (cyproconazole, epoxiconazole, flusilazole, tebuconazole, and prochloraz) as well as pesticides from different chemical classes like chlorpyrifos (an organophosphate) and triflusulfuron-methyl (a triazinylsulfonylurea herbicide) on steroid hormone production and gene expression in a human placental cell line (Jeg-3). The findings demonstrated dose-dependent inhibition in progesterone production by the triazoles as well as the azole fungicide prochloraz. In contrast, the non-triazoles, chlorpyrifos and triflusulfuron-methyl, did not impact progesterone production or show additive effects when mixed with prochloraz. Although prochloraz slightly increased aromatase expression and estradiol production, none of the substances affected the expression levels of steroidogenic cytochrome P450 enzymes. For some of the triazoles, prochloraz, and chlorpyrifos, there was significant induction of CYP1A1 mRNA expression, and combination effects were noted for this endpoint. The inhibition of CYP1A1 mRNA induction by the AhR inhibitor CH223191 suggested that this effect was mediated via the aryl hydrocarbon receptor (AhR). The results underline the importance of evaluating both single and combination effects of pesticides, as they can produce additive interactions affecting hormonal regulation and enzymatic processes in human placental cells.

**Fig 2:**
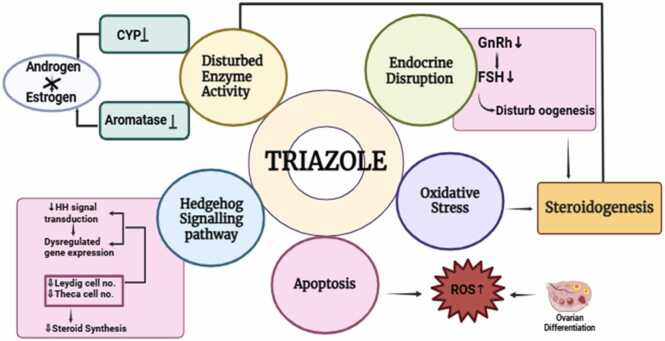
Triazole mediated reproductive toxicity: Mechanism of action. Created with BioRender https://www.biorender.com.

### 2.1 Mechanism of toxicity

Triazoles follow a number of mechanisms to induce adverse impact on female reproductive system and development of embryo. Few of them listed below –

#### 2.1.1 Alteration of enzyme activity

Cytochrome P450 enzymes are crucial for metabolizing a wide variety of substrates, such as medications, environmental pollutants, and endogenous substances. It is primarily located in the ovary and liver. It facilitates the oxidation of substances, converting them into more water-soluble forms for easier excretion. Additionally, Cytochrome P450 enzymes are essential for the synthesis of steroid hormones, such as sex hormones and corticosteroids, and contribute to the production of other important molecules like vitamin D and eicosanoids. Their diverse functions make them vital in maintaining biochemical balance and protecting the body from harmful substances [48]. In the ovary, cytochrome P450 enzymes play a crucial role in the synthesis and metabolism of steroid hormones, such as estrogen, progesterone, and androgens. Proper P450 enzyme activity is vital for optimal ovarian function and fertility. Cytochrome P450 aromatase (CYP19), facilitates the production of estrogens which are essential for regulating ovarian functions like follicle development, ovulation, and corpus luteum maintenance. Additionally, they help in metabolizing and detoxifying hormones to maintain hormonal balance and reproductive health [49].

Many triazoles, particularly antifungal agents like fluconazole and voriconazole, work by inhibiting specific cytochrome P450 (CYP) enzymes in fungal cells which is responsible for the synthesis of ergosterol, a key component of the fungal cell membrane. When triazoles inhibit these enzymes, they disrupt ergosterol production, which weakens the cell membrane and leads to fungal cell death. While this is the desired effect in antifungal therapy, it can also affect the function of human CYP enzymes, potentially leading to drug-drug interactions and hepatotoxicity (liver toxicity).

Triazoles can also affect the activity of enzymes involved in hormone metabolism and signaling pathways. Changes in enzyme activity might influence the regulation of ovarian function and folliculogenesis. Triazoles can inhibit human CYP enzymes, particularly CYP3A4 and CYP2C9, which in turn can affect steroidogenesis in females. Serra *et al*. [16] has also supported the disrupted steroidogenesis in granulosa cells via suppressed expression of CYP51, STAR, CYP11A1, CYP19A1, or HSD3B proteins. Some triazoles have been shown to inhibit aromatase, an enzyme involved in the conversion of androgens to estrogens. This inhibition could lead to decreased estrogen levels, potentially disrupting hormonal balance [50].

The aryl hydrocarbon receptor (AHR) is crucial in regulating ovarian function, affecting the number and growth of antral follicles, steroid hormone production, and ovulation. AHR influences follicular steroidogenesis, potentially by modulating the steroidogenic pathway at multiple stages. It also plays an important role in controlling apoptosis of oocytes in germ cell nests during embryonic development and regulates the survival of oocytes in both the fetal and neonatal ovary. Both the deletion of AHR and the activation of its pathway can impact steroidogenesis in the ovary [51]. Several fungicides, including prothioconazole (PT), epoxiconazole (Epox), tetraconazole (TT), tebuconazole (TB), difenoconazole (Dif), cyproconazole (Cypro), and metconazole (MC), may impair steroidogenesis, possibly by increasing AHR expression in chicken testis [52] and human granulosa cells [53].

#### 2.1.2 Endocrine disruption

The female reproductive cycle is highly coordinated via hypothalamic–pituitary–ovarian axis. Gonadotropin-releasing hormone (GnRH) is an important regulator in this process, as it stimulates the adenohypophysis to release both follicle-stimulating hormone (FSH) and luteinizing hormone (LH). FSH promotes the development of ovarian follicles, activates aromatase in granulosa cells for estrogen production, and stimulates LH receptors in both the corpus luteum and these cells. The peak surge of LH induces ovulation, and the corpus luteum, formed afterward, produces progesterone. Disruption of this finely tuned system by endocrine disruptors at any level can interfere with ovarian function and folliculogenesis, potentially leading to fertility issues and reproductive health problems. Triazoles are classified as endocrine-disrupting chemicals (EDCs), meaning they can interfere with the endocrine system, impacting hormone synthesis, secretion, transport, binding, action, or elimination. As a result, triazoles may alter the production or function of reproductive hormones, causing imbalances that impair ovarian function and fertility [54].

Reductions in a cholesterol carrier (STAR), an enzyme involved in de novo cholesterol synthesis (CYP51), and three essential steroidogenesis enzymes (CYP11A1, HSD3B, and CYP19A1) was noted by Serra *et al*. [16].

Wang *et al*. [17] examined the transcriptomic changes in HepG2 (hepatoblastoma cell line) cells exposed to five triazole fungicides—hexaconazole (HEX), tebuconazole (TEB), propiconazole (PRO), cyproconazole (CYP), and difenoconazole (DIF)—and found that these fungicides significantly impacted estrogen signaling pathways. They observed that the fungicides caused abnormal estrogen secretion by activating the estrogen receptor alpha (ERα) and disrupted key genes involved in estrogen synthesis, including StAR, CYP11A1, 3βHSD2, CYP17, CYP19, CYP3A4, CYP1A2, and SCP2. This disruption affected the metabolism of macromolecules like lipids, amino acids, and carbohydrates, as well as signal transduction. Additionally, exposure to epoxiconazole led to a decrease in progesterone (Pg) and estradiol (E2) production, and reduced mRNA expression of important steroidogenesis enzymes (STAR, HSD3B, and CYP19A1) at a concentration of 25 µM [53].

#### 2.1.3 Signaling pathway disruption

Normal functioning of various signaling pathways, including Wingless-like, the Hedgehog pathways are required for embryonic development, tissue homeostasis, and a variety of physiological processes in both vertebrates and invertebrates. Triazoles have been reported to disrupt signaling pathways, and consequently negatively affecting female reproductive function [17].

The Hedgehog pathway regulates cell differentiation, tissue patterning, and stem cell maintenance. Hedgehog signaling is necessary during embryonic development to form various tissues and organs. It affects neural tube patterning, limb development, and cell type differentiation. Disruptions in Hedgehog signaling during embryogenesis can result in severe developmental defects [55]. Normal ovarian development requires coordinated communication between oocytes, granulosa cells, and theca cells. Granulosa cells produce two Hedgehog (Hh) pathway ligands, Desert hedgehog (Dhh) and Indian hedgehog (Ihh), which work together to regulate the specification and development of theca cells. Liu *et al*. [56] found that female mice with a double knockout of Dhh/Ihh were infertile due to the absence of theca cells and their androgen production. However, single-knockout mice for Dhh or Ihh remained fertile with normal folliculogenesis, although they showed reduced androgen production and changes in their ovarian transcriptomes. Antifungal drugs like itraconazole can disrupt Hedgehog (HH) signaling by altering the expression of downstream components, including Ihh, Gli1, Ptch1, and Smo, which can negatively impact ovarian development in rats [19].

WNTs are highly conserved signaling molecules that regulate critical processes such as cell proliferation, differentiation, fate specification, embryonic induction, and the establishment of cell polarity, through both β-catenin-dependent and β-catenin-independent pathways. *In vitro* transcriptomic analysis revealed that difenoconazole (DIF) suppressed the Wnt signaling pathway, significantly increasing the phosphorylation of β-catenin and altering the expression of related genes in zebrafish embryos. Additionally, exposure to DIF activated PPARγ while inhibiting the Wnt/β-catenin signaling pathway [57].

#### 2.1.4 Oxidative stress

Certain azole fungicides may cause oxidative stress by producing reactive oxygen species (ROS). Oxidative stress can damage cellular structures and macromolecules, resulting in developmental defects. Male and female reproductive dysfunctions are mostly mediated by oxidative stress [58], [59], [60]. Exposure to fungicides leads to significant increases in reactive oxygen species (ROS), which in turn causes cellular damage by affecting lipids, proteins, and DNA [61], [62].

Exposure to antifungal agent’s ketoconazole, miconazole, and prochloraz led to enhanced oxidative stress, a significant reduction in GSH levels, and disruptions in mitochondrial function and apoptosis in mouse Sertoli TM4 cells [63].

#### 2.1.5 Apoptosis

Apoptosis plays a crucial role during embryonic development, tissue homeostasis, and immune system regulation. Triazole fungicide can cause apoptosis through ROS-dependent pathway, ER stress, or activation of the mitochondrial pathway and can increase embryolethality and axial skeleton malformations [64].

Tebuconazole (TEB) is a triazole fungicide that is frequently used and works well to treat fungal infections. It has been shown to exert toxic effects on HCT116 cells by triggering apoptosis, which occurs through the generation of reactive oxygen species (ROS)-induced endoplasmic reticulum (ER) stress and activation of the mitochondrial apoptotic pathway [65].

Khwanes *et al*. [66] reported that difenoconazole upregulated the testicular transcripts of Bax and caspase-3 and induced apoptosis.

## 3 Conclusions

In conclusion, this review delves into the effects of triazole compounds on female reproductive health, offering valuable insights into the potential risks and mechanisms linked to exposure during critical periods such as pregnancy and embryonic development. The literature reveals that the specific type of triazole, along with the dosage and exposure duration, plays a crucial role in determining the extent of reproductive effects. Addressing the reproductive risks posed by triazoles requires collaborative efforts between women’s health providers, reproductive specialists, and support staff, alongside proactive measures from local governments, environmental agencies, and policymakers.

## CRediT authorship contribution statement

**Pandey Dr Geeta:** Writing – review & editing, Supervision. **Sharma Sonal:** Writing – review & editing, Writing – original draft.

## Declaration of Competing Interest

The authors declare the following financial interests/personal relationships which may be considered as potential competing interests: Dr. Geeta Pandey reports was provided by IIS (Deemed to be University) Jaipur. Dr Geeta Pandey reports a relationship with IIS (Deemed to be University) Jaipur that includes: employment. If there are other authors, they declare that they have no known competing financial interests or personal relationships that could have appeared to influence the work reported in this paper.

## Data Availability

Data will be made available on request.
